# Exploiting Interlimb Arm and Leg Connections for Walking Rehabilitation: A Training Intervention in Stroke

**DOI:** 10.1155/2016/1517968

**Published:** 2016-06-23

**Authors:** Taryn Klarner, Trevor S. Barss, Yao Sun, Chelsea Kaupp, Pamela M. Loadman, E. Paul Zehr

**Affiliations:** ^1^Rehabilitation Neuroscience Laboratory, University of Victoria, Victoria, BC, Canada V8W 3N4; ^2^Human Discovery Science, International Collaboration on Repair Discoveries (ICORD), Vancouver, BC, Canada V5Z 1M9; ^3^Centre for Biomedical Research, University of Victoria, Victoria, BC, Canada V8W 2Y2; ^4^Division of Medical Sciences, University of Victoria, Victoria, BC, Canada V8W 2Y2

## Abstract

Rhythmic arm and leg (A&L) movements share common elements of neural control. The extent to which A&L cycling training can lead to training adaptations which transfer to improved walking function remains untested. The purpose of this study was to test the efficacy of A&L cycling training as a modality to improve locomotor function after stroke. Nineteen chronic stroke (>six months) participants were recruited and performed 30 minutes of A&L cycling training three times a week for five weeks. Changes in walking function were assessed with (1) clinical tests; (2) strength during isometric contractions; and (3) treadmill walking performance and cutaneous reflex modulation. A multiple baseline (3 pretests) within-subject control design was used. Data show that A&L cycling training improved clinical walking status increased strength by ~25%, improved modulation of muscle activity by ~25%, increased range of motion by ~20%, decreased stride duration, increased frequency, and improved modulation of cutaneous reflexes during treadmill walking. On most variables, the majority of participants showed a significant improvement in walking ability. These results suggest that exploiting arm and leg connections with A&L cycling training, an accessible and cost-effective training modality, could be used to improve walking ability after stroke.

## 1. Introduction

Body weight supported treadmill training therapy can be used for the recovery of walking after neurological damage. In this rehabilitation paradigm, participants walk on a motorized treadmill with a harness system allowing the weakened leg muscles to be freed from the necessity of body weight support and stepping is performed with the help of robotic interfaces or therapists. This protocol was initially utilized after spinal cord injury and may be equally beneficial for recovery of walking after stroke [1–5].

Results from this therapy are positive, but there are significant limitations that limit access for the broader stroke population. Body weight supported treadmill training therapy has significant labour requirements, requires specialized equipment, and is typically only available in restricted environments such as in rehabilitation centers [6, 7]. In addition, body weight supported treadmill training offers no additional benefit over conventional physical therapy, as demonstrated in a large randomized clinical trial [2]. A more cost-effective and generally accessible protocol based upon a device (e.g., arm and leg ergometer or a recumbent stepper) that could be more readily used in therapy would be of great benefit where less training is required for physical therapists to supervise training and participants may be more likely to comply with a community-based training regimen [2, 8].

In addition to finding a rehabilitation program that is widely accessible, exploiting the neural and mechanical linkages between the arms and legs that are inherent parts of human locomotion could enhance the recovery of walking [6, 9, 10]. Therefore, incorporating rhythmic arm movement paradigms for locomotor rehabilitation, such as with arm and leg (A&L) cycling, could be very beneficial to stroke locomotor recovery. Although there are differences in kinematics, balance requirements, and loading of the arms between walking and A&L cycling, this type of training activates similar neural networks that are engaged during walking [11]. We have recently shown that, even following a stroke, neural commonalities between A&L cycling and walking persist, despite altered descending supraspinal input from the stroke lesion [12]. Given that A&L cycling and walking share common neural elements and that this persists following stroke, there is a reasonable basis for expectation of training transfer to improve walking.

The extent to which A&L cycling training can lead to training adaptations which transfer to improved walking function remains untested. Thus, the objective of this project was to test the efficacy of A&L cycling training to enhance walking after stroke. Given that A&L cycling and walking share a common core of subcortical regulation, we hypothesize that A&L cycling training will transfer to an improvement in walking. Improvements in walking function were gauged by changes in clinical walking status, strength, and walking performance. If indirect training with A&L cycling does improve walking function, this adjunct therapy could be used as an additional modality to improve walking ability after stroke.

## 2. Materials and Methods

### 2.1. Participants

Participant recruitment occurred through community stroke support groups, posters in medical offices/hospitals, and newspaper articles. As for inclusion criteria, participants were required to be a minimum of six months after infarct, after spontaneous poststroke changes are thought to have occurred [13], and able to stand free without assistive devices. Participants were screened with the Physical Activity Readiness Questionnaire to determine eligibility to participate in physical activity. If a response of “yes” was given for any of the questions in the questionnaire, indicating the presence of bone or joint problems or dizziness, written medical permission was obtained for that participant. A list of current medications was also obtained for each participant. Exclusion criteria included medications affecting muscle tone less than three months priorly and self-report of any cardiovascular, musculoskeletal, respiratory, or other chronic diseases. A sample size of twenty-five participants was recruited, in line with statistical reports that a sample size of 25 will specify a power of 0.80 at a large effect size and criterion value of *p* = 0.05 [14]. Sample size was based on previous studies of locomotor studies after stroke and other interventions yielding strength gains after stroke [15, 16].

To assist with determining participant's functional status, clinical assessments were performed by a licensed physical therapist. Muscle tone was measured using the Modified Ashworth Scale (5 points) at the ankle and knee for the lower limb [17, 18]. This is a graded rating of spasticity scored from 0 to 4, with 0 being flaccid and 4 being rigid. A measure of the basic motor skills necessary for functional ambulation was derived using the 6-point Functional Ambulation Categories Scale, where level 1 indicates that a patient is nonambulatory and level 6 indicates a patient is fully independent [19]. To measure general physical impairment, the Chedoke-McMaster Stroke Assessment [20] was used. Impairment in the arm (A), hand (H), leg (L), and foot (F) was determined using the 7-point activity scale, where a score of 1 represents complete independence and a score of 7 represents total assistance. Using the 5-piece Semmes-Weinstein kit of calibrated monofilaments (Sammons Preston Rolyan, Cedarburg, WI), ability to discern light touch and pressure was measured in the more affected hand and foot [21]. Reflexes obtained using a reflex hammer were graded on a 0 to 4+ scale, where 0 means a reflex is absent and 4+ represents a hyperactive reflex with clonus for hip flexion (L1) and ankle plantarflexion (S1) [22].

### 2.2. Ethics Statement

The authors confirm that all ongoing and related trials for this intervention are registered (ClinicalTrials.gov: NCT02316405). Informed written consent from each participant was obtained for a protocol approved by the University of Victoria Human Research Ethics Committee (Protocol number: 07-480-04d) and performed according to the Declaration of Helsinki. The study protocol that was registered was the same as the study protocol approved by the University of Victoria Human Research Ethics Committee prior to subject enrollment.

### 2.3. Training Protocol

Participants performed training three times a week, with 30 minutes of aggregate activity time per session, for a total duration of five weeks. Most participants completed training on Monday, Wednesday, and Friday. All experimental and training sessions took place in the Rehabilitation Neuroscience Laboratory at the University of Victoria.

For training, an arm and leg cycling ergometer with coupled upper and lower cranks was used (Sci-Fit Pro 2 ergometer). Dependent motion of the cranks for the arms and legs allows for passive assistance of weaker limbs during training. Mechanical modifications were made to the cycle ergometer to ensure a customized and comfortable fit for each training session. The cranks of the arm and leg ergometer were individually adjusted to the range of motion for each limb of each stroke participant and hand braces were worn when needed to ensure grip on the handle with the more affected (MA) hand. During each session, participants were allowed to take short 1-2-minute breaks during training if required, but the aggregate time for each session was always met. In fact, few participants took breaks and those who did only needed them in the early days of training. Participants were expected to tolerate the protocol very well as this was a modification of a previous protocol where chronic stroke participants performed four trials of six-minute bouts (totalling 24 minutes) of active A&L cycling [16].

To evaluate the physiological cost of training activity, heart rate (HR), rating of perceived exertion (RPE), and revolutions per minute (RPM) were collected every five minutes. Heart rate was monitored with a chest strap heart rate monitor (Polar Electro, Quebec, Canada) and recorded in beats per minute (bpm). The rating of perceived exertion was self-reported using the 10 pt scale [23]. A&L cycle ergometer RPM were recorded visually from the digital display on the cycle ergometer as participants used this signal as a source of visual feedback for maintaining cadence. A single value for each variable was created each session by individually averaging the HR, RPE, RPM, and Watts over the 30 minutes of training and differences between the first and last training session were inspected.

Participants were encouraged to exercise at a moderate level sufficient to report a RPE value between three and five, corresponding to a target heart rate between 50 and 70%  HR_max_ [24]. Target heart rate training zones were calculated with the Karvonen Formula taking into account heart rate reserve, and, if a participant reported being on beta blockers, adjustments to target heart rate goals were made [25]. The progressive element of this steady-state training included increasing the resistance of the ergometer over five weeks in order to maintain the same relative RPE. This is in line with many other poststroke treadmill training protocols where training volume was increased [26]. Increases in resistance were only required for 6 out of 19 participants and generally increases were made in 5 W increments to a maximum of 40 W. During the training and testing time, participants were also encouraged to maintain their normal activity levels but to not participate in additional research programs or interventions.

All exercise sessions were supervised by a Certified Exercise Physiologist with the Canadian Society for Exercise Physiology as well as several laboratory assistants to ensure appropriate monitoring. Exercise sessions were not initiated if participant's blood pressure exceeded 140/90 mmHg in accordance with Canada's Physical Activity Guidelines [27]. Exercise was terminated if HR exceeded 85% of the age-predicted maximum, if blood pressure exceeded 200/110 mmHg, and if the participant felt dizzy, nervous, or pain in the chest. Upon completion of the 30 minutes in each training session, participants were given three to five minutes to cool down and remained in the laboratory until blood pressure returned to preexercise values. All blood pressure values were obtained with a digital blood pressure cuff placed over the less affected arm.

### 2.4. Multiple Baseline and Posttest Measures

**Figure 1:**
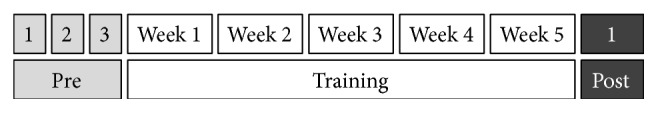
Illustration of the testing and training protocol. A multiple baseline within-subject control design was used for this study.

A multiple baseline within-subject control design was used for this study [28, 29].  Figure 1 illustrates the testing and training protocol. A multiple baseline design allowed for the creation of a reliable and consistent pretest measure, allowed for inspection of spontaneous recovery effects, and provided baseline data against which changes were evaluated. In this design, the control group is the experimental group. Multiple baseline measurements were obtained from participants in three baseline experimental sessions over a period of three to four weeks, with at least six days between baseline sessions. The posttest following training was performed within three days following training. As it was impossible to blind participants in this trial, several things were done to help control for potential sources of bias. At experimental sessions, the same tests were performed in the same order and environmental conditions (i.e., temperature, noise, lighting, and participant position) and session time of day were kept as consistent as possible [15, 30, 31]. These measures have been previously shown to have high reliability across multiple baseline points [29]. The project manager, who was in charge of participant recruitment and scheduling, did not take part in the assessment of outcomes, nor did the exercise supervisors. Analysis of data was mainly performed by laboratory assistants who were not involved in the design or interpretation of results.

### 2.5. Clinical Walking and Balance Measures

Clinical assessment of walking was performed by the same licensed physical therapist who was not involved in the study both before and after intervention. Tests included the Timed Up and Go test [32], timed 10 m walk test [33], and the 6-minute walking test [34]. These clinical walking tests assessed overground walking mobility, speed, and endurance. Balance was also assessed before and after intervention with the Berg Balance Scale [35].

### 2.6. Strength and Muscle Activation (EMG)

Maximal voluntary isometric contractions were assessed for ankle dorsiflexion, plantarflexion, and handgrip, measured bilaterally. Similar to previous studies [15, 36], participants were assessed while seated in a custom-fit chair designed to minimize movement. Maximum forces produced during dorsiflexion and plantarflexion contractions were established via strain gauge (Omegadyne Ltd. Model 101-500) and converted to torque using a moment arm length of 0.15 m (measured from the heel block to the center of the strain gauge). Hand grip was performed with a commercial dynamometer (Takei Scientific Instruments Company Ltd., Niigata, Japan). In 10-second trials, following a silent period of 5 seconds, contractions were held for each limb separately. Following a brief warm-up, participants were given two attempts for achieving a maximum contraction.

Electromyographic (EMG) data from the soleus (SOL), tibialis anterior (TA), flexor carpi radialis (FCR), and posterior deltoid (PD), from the more affected (MA) and less affected (LA) limbs, were collected with surface electrodes placed in bipolar configuration over the muscle bellies of interest. Electrodes were placed on the skin and oriented longitudinally along the fibre direction, in accordance with SENIAM procedures [37]. Electrodes on the upper and lower limbs were placed in the same positions at each testing session. This was accomplished by recording cathode and anode electrode distances from anatomical landmarks and with pictures taken at the first session and the electrodes were placed by the same experimenter each time. As with other studies from this laboratory [11, 42], EMG signals were preamplified (×5000) and band-pass filtered (100–300 Hz) (Grass P511, Astro-Med). Data were converted to a digital signal, sampled at 1000 Hz using custom built continuous acquisition software (LabVIEW, National Instruments, TX, USA) and stored to a PC for offline analysis. Using custom-written software programs (Matlab, The Mathworks, Inc., MA, USA), EMG data were full-wave rectified and low-pass filtered at 6 Hz using a 4th-order Butterworth filter to obtain the liner envelope. Maximum values were taken as the greatest reading generated over two trials by obtaining the mean value over 500 ms when force and EMG signals were highest.

### 2.7. Walking

Similar to previously reported methods [38], participants walked at a self-selected (“comfortable”) speed on a motorized treadmill (Woodway USA, Waukesha, WI) while wearing an overhead safety harness (Pneu-Weight, Pneumex Inc., Sandpoint, ID, USA). All participants wore the safety harness without body weight support both before and after intervention and none wore an ankle foot orthosis. Participants were free to use hand-held railings in front or beside them during the trial and arm position did not change between pretests and the posttest. The self-selected treadmill speed (0.51 ± 0.32 mph) was held constant for that participant for baseline and posttraining tests to control for the effects of change in treadmill speed with changes in EMG [43].

EMG data for walking was collected in a similar manner as for strength but was normalized to maximal EMG recorded during walking. To quantify the rhythmic activation of muscles during walking, a modulation index (MI = [(EMG_max_ − EMG_min_)/EMG_max_] × 100) was calculated for each muscle across each movement cycle and averaged. This measure provides a means of comparing the extent to which muscles varied from phasic bursts of activity to alternatively tonic activity throughout the movement cycle [38, 39, 44, 45]. This measure provides an index of overall amplitude modulation across the movement cycle. Higher values, closer to 100%, indicate a larger range of modulation for a muscle with periods of contraction and periods of relaxation, while a lower value indicates that muscle's activity is more constant [45].

To detect joint kinematics, goniometers (Biometrics Inc., Ladysmith, VA) were used for both the LA and MA ankle (dorsiplantarflexion) and knee (flexion/extension). These devices were calibrated, output in degrees was determined, and data were sampled at 1000 Hz. Kinematic data were low-pass filtered at a cut-off frequency of 6 Hz with a fourth-order dual-pass Butterworth filter and were quantified by determining the range of motion by calculating the maximum and minimum angular excursions recorded through the stride cycle.

Similar to other studies [38, 46–49], custom-made force sensing resistors (FSR) (model 1027-1001-ND, Digi-Key, Thief River Falls, MN, USA) were inserted into both shoes under the heel and first metatarsal head of each foot. Heel-contact could not be precisely determined as there was some impairment in heel-strike for these participants; therefore, FSR signals from the foot sole were summed and used to define stride cycles as periods of stance (foot contact) and swing (no foot contact). The average duration between the starts of ipsilateral foot-contacts, duration of stance, and duration of swing were determined. Stride frequency was determined as the average number of strides taken in one second. EMG and kinematic data for the LA and MA sides were aligned to begin with foot contact for that respective side.

### 2.8. Cutaneous Reflexes

The pattern of cutaneous reflex modulation during walking was used to assess the strength of adaptation arising from A&L cycling training. Cutaneous reflexes were evoked via combined surface stimulation of the nerves innervating the dorsum of the hand (superficial radial; SR) and foot (superficial peroneal; SP) [11]. Electrodes for SR and SP nerve stimulation were placed just proximal to the radial head and on the crease of the ankle, respectively, on the LA limbs. A Grass S88 stimulator with SIU5 stimulus isolation and a CCU1 constant current unit (Astro-Med Grass Instrument, West Warwick, RI) were used to deliver stimulation in trains of 5 × 1.0 ms pulses at 300 Hz (P511 Astro-Med Grass Instrument). Perceptual and radiating thresholds (RT) were determined and nonnoxious intensities were found for each participant. Stimulation intensities were set to 2.2 × RT for the SR nerve and 2.0 × RT for the SP nerve. During treadmill walking, 120 stimulations were delivered pseudorandomly with an interstimulus interval of 1–5 seconds.

All data were sampled at 1 kHz with a 12-bit A/D converter connected to a computer running custom-written LabVIEW (National Instruments, Austin, TX) virtual instrument applications. Evoked reflexes in all muscles tested were aligned to delivery and averaged together. The stimulus artefact was removed from the reflex trace and data were then low-pass filtered at 30 Hz using a dual-pass, fourth-order Butterworth filter. To investigate phase-dependent modulation within each movement cycle, data were broken apart into 8 equally timed phases with phases 1–5 representing LA stance and phases 6–8 representing LA swing for walking [11]. For reflexes within each phase, the average trace from the nonstimulated data was subtracted from the stimulated average trace to produce a subtracted EMG reflex trace. Cutaneous reflexes were quantified as the average cumulative reflex over 150 ms following stimulation within each of the 8 phases [48, 50]. Background EMG levels between tests were also compared to inspect for a possible scaling effect on reflex activity. A modulation index (MI) for change in reflexes relative to maximum background activity (bEMG) across phases for each muscle was also calculated (MI = [(Reflex_max_ − Reflex_min_)/bEMG_max_] × 100).

### 2.9. Statistics

Using commercially available software (SPSS 18.0, Chicago, IL), pretest and posttest data were compared. Using commercially available software (SPSS 18.0, Chicago, IL), pretest and posttest data were compared. To evaluate the extent to which arm and leg cycling training altered walking ability, posttest data were compared to the 95% confidence interval (CI) created from three pretest sessions and compared to a pretest average for each participant. To establish the 95% CI for each measure, variability was computed from 3 pretest sessions and used to create a data range with which the posttest value was compared. If the posttest value fell outside the 95% CI range, it was considered significant for that participant. The total number of participants with a significant test is reported and dichotomous scores (1 representing a posttest score outside of the 95% CI range and 2 representing a score within the 95% CI range) for each participant for each measure were compared with the chi-squares test statistic to examine association.

For pretest data, a repeated-measures ANOVA was performed to examine difference across the three pretest sessions. If no difference was found, data were pooled together to create an average pretest value and compared to posttests values with paired-samples *t*-test (*t*). For each test, the degrees of freedom are reported in subscript. Assumptions for ANOVA and paired-samples *t*-tests were evaluated for parametric tests for a within-subject design. Pearson's correlation coefficients (*r*) were calculated between several variables and tested for significance. The observed effect for posttest differences for clinical measures, strength, and walking parameters is also reported as Cohen's effect size (*d*), where a small effect is *d* = 0.2, a medium effect is *d* = 0.5, and a large effect is *d* = 0.8 [51]. For priori hypotheses where direction of change was predicted, one-tailed paired-samples *t*-tests were performed. Statistical significance was set at *p* ≤ 0.05.

## 3. Results

A total of 25 participants were recruited. Six participants were excluded because of self-withdrawal (*n* = 1), change in physical activity patterns (*n* = 1), and not meeting minimum inclusion criteria or were already participating in A&L cycling exercise (*n* = 4). Baseline and demographic data are reported for the remaining 19 participants (see Table 1). All participants contributed data to each measure.

### 3.1. Training Results

**Figure 2:**
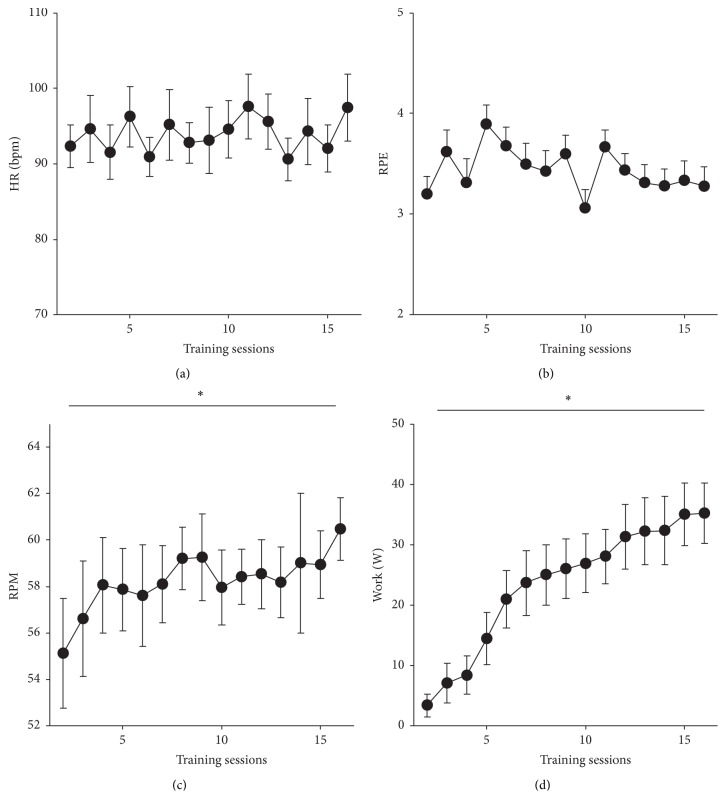
Training parameters for HR, RPE, RPM, and Work over 15 training sessions. Data are means (±sem) averaged across all participants for all training sessions. ^*∗*^indicates a significant difference between first and last training sessions.

All participants completed the 15 sessions of A&L cycling training.  Figure 2 shows the average HR, RPE, RPM, and Work in each of the 15 training sessions averaged across all participants. Within a session, HR increased between minute 5 and minute 30 from 76.0 ± 1.9 bpm to 98.9 ± 3.1 bpm and there was no significant difference between the first training session and the last training session. Across sessions, while there was no change in HR and RPE, there was a significant increase in RPM (*t*_(18)_ = 2.399 and *p* = 0.014, Figure 2(c)) and Work (*t*_(18)_ = 6.475 and *p* = 0.000, Figure 2(d)) between the first and last training session. Despite increases in RPM and Work, the same relative RPE was maintained.

### 3.2. Clinical Measures

A paired *t*-test revealed that there was a significant decrease (14.4% change (*t*_(18)_ = 2.100, *p* = 0.025, and *d* = 0.350)) in the time taken for the Timed Up and Go test where participants completed the test in 29.33 ± 25.83 seconds before training and 25.12 ± 22.14 seconds after training. Time taken for the 10 m walk test also significantly decreased where participants completed the test at 0.45 ± 0.50 m/sec before training and 0.51 ± 0.48 m/sec after training indicating a 13.3% improvement (*t*_(18)_ = 2.342, *p* = 0.015, and *d* = 0.192). The number of steps taken for the 10 m walk test also significantly decreased with 27.17 ± 12.44 steps before training and 25.69 ± 12.50 steps after training indicating a 5.45% improvement (*t*_(18)_ = 2.140, *p* = 0.023, and *d* = 0.239). The total distance covered in the 6-minute walk test significantly increased between the pre- and posttest from 217.41 ± 107.67 feet to 252.43 ± 138.38 feet indicating a 16.10% improvement (*t*_(18)_ = 3.586, *p* = 0.001, and *d* = 0.564). The total score from the Berg Balance Scale significantly increased following A&L cycling training from a mean score of 42.04 ± 10.48 to a mean score of 45.06 ± 2.38 (median scores of 45 to 48 after training) indicating a 4.94% improvement (*t*_(18)_ = 2.825, *p* = 0.005, and *d* = 0.528).

Table 2 summarizes results from the single-participants statistical tests that are discussed below. The number of participants with a significant posttest value is reported for each variable in the table. For most variables, the majority of participants did show a significant posttest change.

### 3.3. Strength and EMG

**Figure 3:**
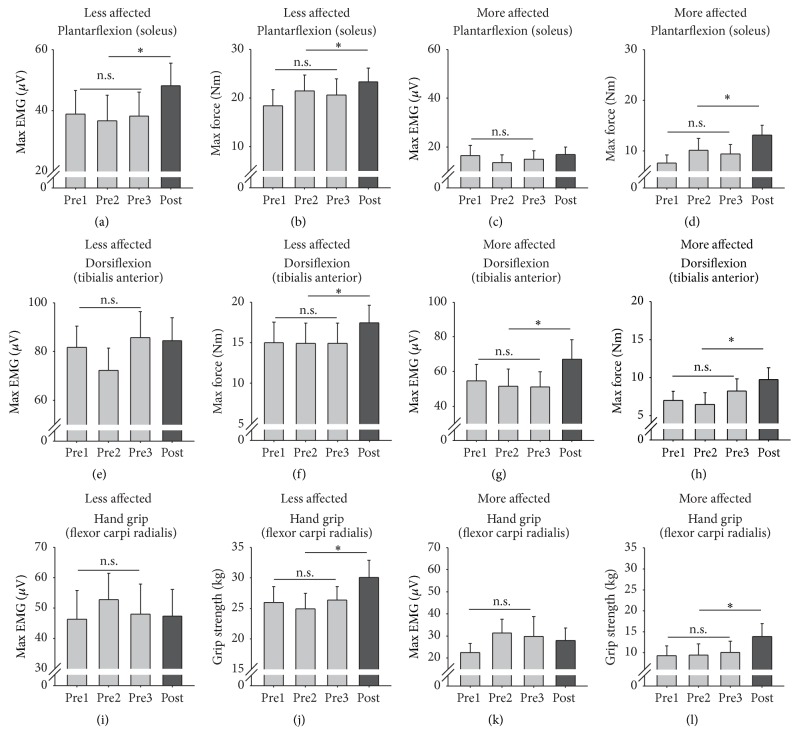
Plantarflexion, dorsiflexion, and hand grip strength and muscle activation. Bar graphs are means (±sem) for EMG and force during isometric strength tests averaged across all participants. ^*∗*^indicates significant differences between the pretest average and the posttest value and n.s. indicates a nonsignificant difference for the three baseline measures.

Figure 3 shows peak EMG activity and force during plantarflexion, dorsiflexion, and handgrip averaged across all participants for three pretests and the posttest conditions. No significant differences were found for pretest baseline data. Following training, plantarflexion force was significantly increased on the LA side by 15.48% and on the MA side by 44.93% (*t*_(18)_ = 2.061, *p* = 0.029, and *d* = 0.437, Figure 3(b) and *t*_(18)_ = 2.073, *p* = 0.029, and *d* = 0.439, Figure 3(d) for the LA and MA sides, resp.). Maximal soleus EMG on the LA side also increased by 27.14% (*t*_(18)_ = 2.154, *p* = 0.025, and *d* = 0.453, Figure 3(a)). The increase in plantarflexion force and SOL EMG on the LA side was significantly correlated (*r* = 0.499 and *p* = 0.045). For dorsiflexion, LA force significantly increased by 16.61% and MA force significantly increased by 34.93% (*t*_(18)_ = 1.821, *p* = 0.045, and *d* = 0.394, Figure 3(f) and *t*_(18)_ = 2.244, *p* = 0.021, *d* = 0.568, and *d* = 1.057, Figure 3(h) for the LA and MA sides, resp.). Peak tibialis anterior EMG also significantly increased on the MA side by 27.91% (*t*_(18)_ = 1.946, *p* = 0.036, and *d* = 0.417, Figure 3(g)). The increase in MA dorsiflexion force and MA TA EMG activity was significantly related (*r* = 0.742 and *p* = 0.001). Handgrip strength significantly increased on the LA side by 16.74% and on the MA side by 44.78% (*t*_(18)_ = 4.010, *p* = 0.001, and *d* = 0.687, Figure 3(j) and *t*_(18)_ = 5.026, *p* = 0.000, and *d* = 0.764, Figure 3(l) for the LA and MA sides, resp.). There was an association between the likelihood of a significant increase in LA strength and MA strength (*χ* ^2^_(1)_ = 23.768 and *p* < 0.0001).

### 3.4. Walking

**Figure 4:**
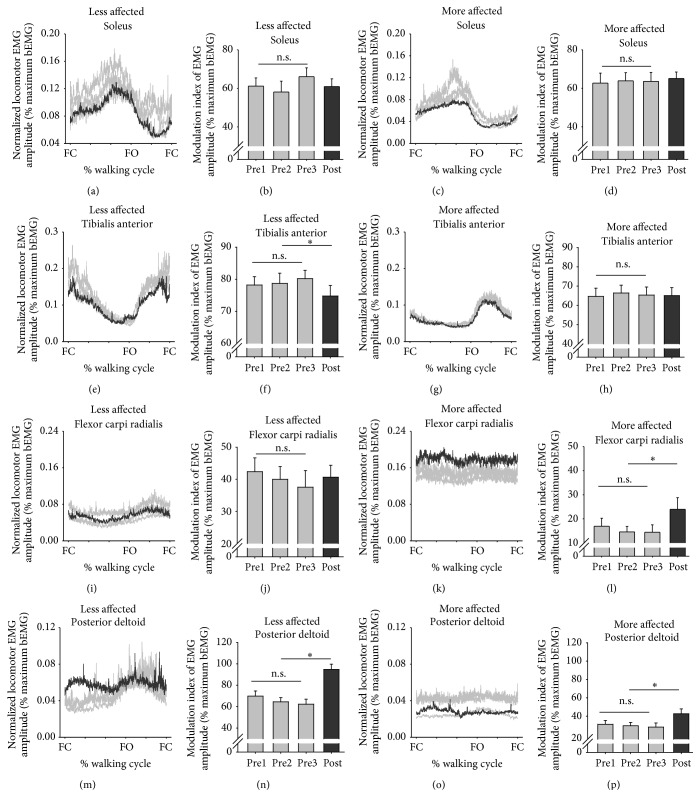
Background EMG during walking. Line graphs are normalized and averaged EMG for the walking cycle for three baseline tests (light gray lines) and for the posttest (dark gray lines). Foot contact (FC) and foot off (FO) times are indicated. Bar graphs are mean (±sem) modulation indices for all muscles averaged across all participants. ^*∗*^indicates significant differences between the pretest average and the posttest value and n.s. indicates a nonsignificant difference for the three baseline measures.

Figure 4 shows EMG for the muscles of the LA and MA limbs averaged across all participants for three pretests and for posttest values during walking. Line graphs are data expressed as a percentage of the gait cycle where 0% indicates foot contact for that side. Bar graphs are background EMG modulation indices across muscles averaged for all participants. No significant pretest differences were found for any muscles. Following training, for the LA TA, there was a significant decrease (*t*_(18)_ = 1.875, *p* = 0.041, and *d* = 0.398, Figure 4(f)) in modulation by 6.4%. In the MA FCR, modulation significantly increased (*t*_(18)_ = 2.134, *p* = 0.027, and *d* = 0.496, Figure 4(k)) by 34.7% and modulation also significantly increased for both the LA and MA PD by 12.1% and 28.9% (*t*_(18)_ = 2.975, *p* = 0.004, and *d* = 0.827, Figure 4(n) and *t*_(18)_ = 2.259, *p* = 0.021, and *d* = 0.649, Figure 4(p) for the LA and MA PD, resp.). When comparing the ratio of modulation between the LA and MA sides for each muscle, there was a significant decrease of 49.2% in ratio for the PD (*t*_(18)_ = 3.085, *p* = 0.009, and *d* = 0.423).

**Figure 5:**
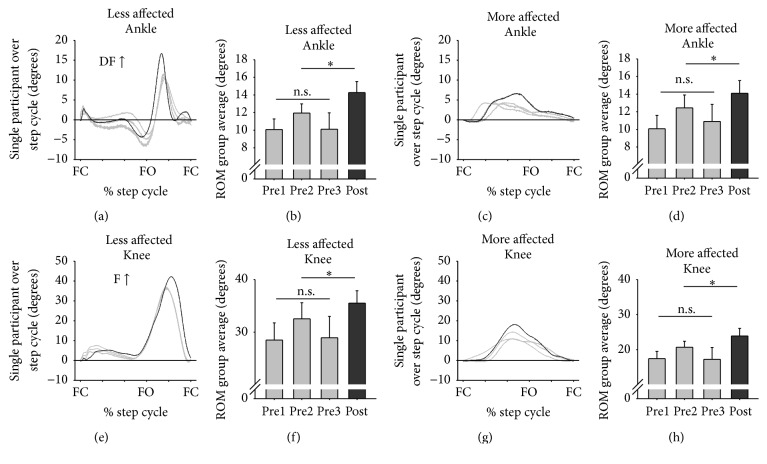
Kinematics during walking. Line graphs are single participant kinematics for the walking cycle for three baseline tests (light gray lines) and for the posttest (dark gray lines). Foot contact (FC) and foot off (FO) times are indicated. Dorsiflexion (DF) and flexion (F) increases are positive. Bar graphs are mean (±sem) range of motion values averaged across all participants. ^*∗*^indicates significant differences between the pretest average and the posttest value and n.s. indicates a nonsignificant difference for the three baseline measures.

Figure 5 shows kinematic data for the LA and MA ankle and knee averaged across all participants for three pretests and for posttest values during walking. Line graphs are from a representative participant aligned to begin at foot contact and bar graphs are ROM values averaged across all participants. No pretest differences were found for any kinematic variables. Following training, all variables showed statistically significant increases in ROM for posttest compared to pretest values (LA ankle: *t*_(18)_ = 2.970, *p* = 0.004, and *d* = 0.558, Figure 5(b); MA ankle: *t*_(18)_ = 2.078, *p* = 0.027, and *d* = 0.426, Figure 5(d); LA knee: *t*_(18)_ = 2.561, *p* = 0.010, and *d* = 0.382, Figure 5(f); and MA knee: *t*_(18)_ = 3.404, *p* = 0.002, and *d* = 0.476, Figure 5(h)). For the ankle, there was a 25.51% increase in ROM for the LA side and a 21.73% increase in ROM for the MA side. For the knee, there was a 19.37% increase in ROM for the LA side and a 22.21% increase in ROM for the MA side. There was a significant association between a change in LA and MA kinematics (*χ* ^2^_(1)_ = 3.979 and *p* = 0.046).

**Figure 6:**
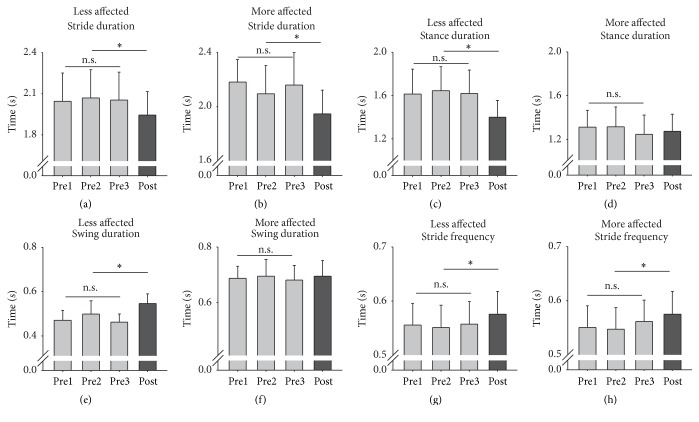
Temporal parameters of walking. Bar graphs are mean (±sem) values for stride duration, stance duration, swing duration, and stride frequency for three baseline tests and the posttest averaged across all participants. ^*∗*^indicates significant differences between the pretest average and the posttest value and n.s. indicates a nonsignificant difference for the three baseline measures.

Walking parameters including average stride, stance and swing durations, and stride frequencies from the LA and MA sides averaged across all participants for the three pretests and for posttest are shown in Figure 6. No baseline differences were detected for any walking parameter data. Following training, there was a significant decrease in stride duration on the LA and MA sides (*t*_(18)_ = 2.448, *p* = 0.013, and *d* = 0.500, Figure 6(a) and *t*_(18)_ = 3.077, *p* = 0.003, and *d* = 0.587, Figure 6(b) for the LA and MA sides, resp.) with a 5.25% and 8.74% decrease in LA and MA stride duration. Stance duration for the LA side significantly decreased (*t*_(18)_ = 2.457, *p* = 0.013, and *d* = 0.501, Figure 6(c)) by 12.53%, while swing duration increased (*t*_(18)_ = 1.837, *p* = 0.042, and *d* = 0.397, Figure 6(e)) by 11.29% following A&L cycling training. There were also significant increases in stride frequency compared to the pretest values for both the LA and MA sides (*t*_(18)_ = −1.961, *p* = 0.033, and *d* = 0.419, Figure 6(g) and *t*_(18)_ = −2.114, *p* = 0.025, and *d* = 0.446, Figure 6(h), for the LA and MA sides, resp.). Stride frequency increased by 3.82% for the LA side and 4.07% for the MA side. Percentage change in stride duration is significantly correlated with percentage change in stride frequency for both the LA (*r* = −0.989 and *p* = 0.000) and MA (*r* = −0.702 and *p* = 0.001) sides. There was a significant association between a change in MA and LA walking parameters (*χ* ^2^_(1)_ = 30.728 and *p* = 0.000). These changes in walking parameters following training were independent of changes in speed as treadmill speed was held constant across all testing sessions.

### 3.5. Cutaneous Reflexes

**Figure 7:**
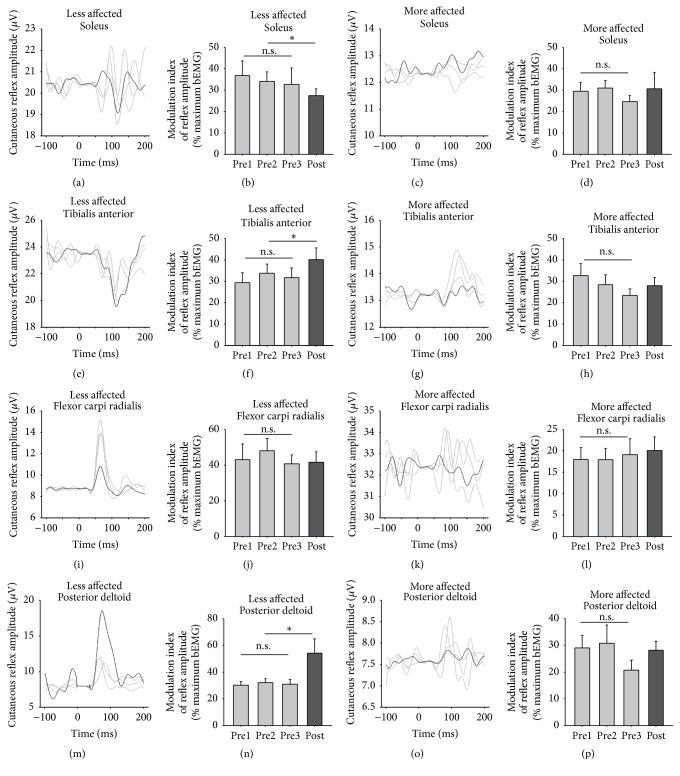
Cutaneous reflexes during walking. Line graphs are averages across all participants for three baseline tests (light gray lines) and for the posttest (dark gray line). The stimulus artefact beginning at time 0 has been blanked out and replaced with a flat line. Stimulation was applied to the superficial radial nerve of the hand and the superficial peroneal nerve of the foot on the LA side. Bar graphs are means (±standard error) averaged across all participants for baseline and posttest values. ^*∗*^indicates significant differences between the pretest average and the posttest value and n.s. indicates a nonsignificant difference for the three baseline measures.

Figure 7 shows data for all reflexes evoked during treadmill walking averaged across all participants. This process reveals the general trend in evoked responses but obscures phase-modulation. To quantify overall modulation of reflexes, a modulation index was quantified for the muscles on the LA and MA sides and shown as bar graphs on Figure 7. Following training, modulation was significantly decreased for the LA SOL (*t*_(18)_ = 2.217, *p* = 0.045, and *d* = 0.355, Figure 7(b)) by 29.3%. For the LA TA, modulation significantly increased by 44.6% (*t*_(18)_ = 3.493, *p* = 0.004, and *d* = 0.378, Figure 7(f)), and, for the LA PD, modulation increased by 80.4% (*t*_(18)_ = 2.197, *p* = 0.047, and *d* = 0.386, Figure 7(n), resp.). There was a significant association between a change in MA and LA cutaneous reflex modulation (*χ* ^2^_(1)_ = 5.793, *p* = 0.016).

**Figure 8:**
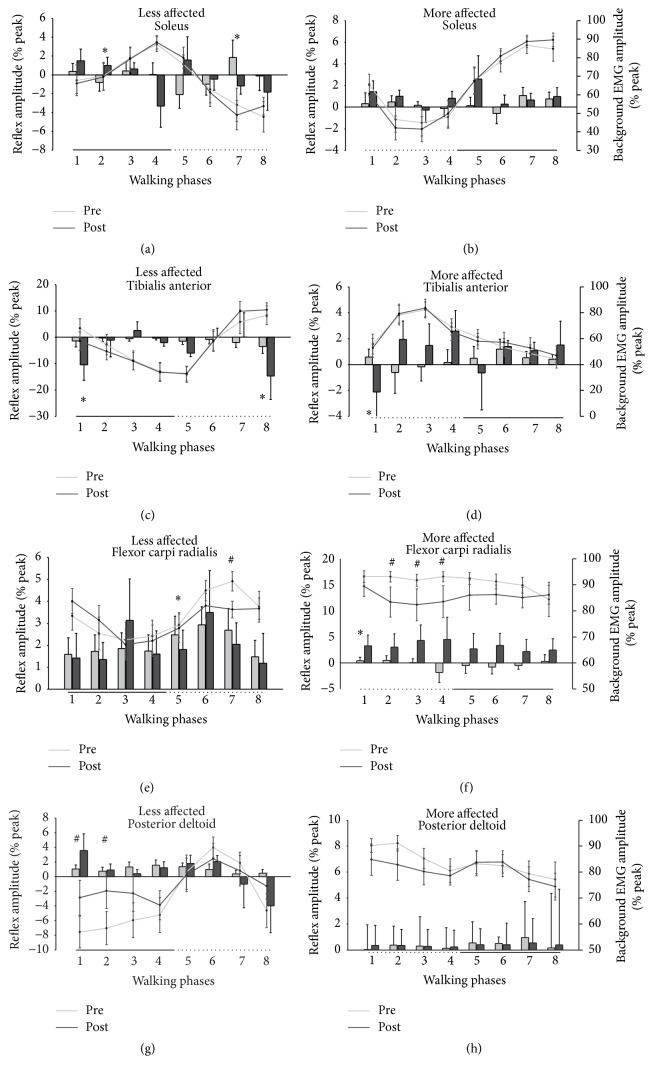
Normalized background EMG and reflex amplitudes during walking. Background EMG is shown in line plots and reflex amplitude is shown in bar plots. Values are means (±standard error) averaged across all participants and normalized to the peak undisturbed EMG during walking. The horizontal bars below the* y*-axes represent the stance (solid line) and swing (dotted line) phase of walking. Significant differences between the pretest average and the posttest value are indicated with # for background EMG and *∗* for reflex amplitude.

Figure 8 shows cutaneous reflexes (bars) during walking at all phases. Since there were no significant differences between the pretest data, for simplification, the average value across the three tests is shown. For reflex amplitudes, there are significant differences between baseline and posttest values for several muscles, including the LA SOL, LA, and MA TA, and for the LA and MA FCR. For the LA SOL, there was a significant change in reflex amplitude for phase 2 (*t*_(18)_ = 2.207 and *p* = 0.046) and phase 7 (*t*_(18)_ = 2.271 and *p* = 0.021). For the LA TA, phase 1 and phase 8 showed significant differences in posttest values compared to the baseline average (for phase 1 *t*_(18)_ = 2.271 and *p* = 0.041 and for phase 8 *t*_(18)_ = 1.871 and *p* = 0.042). For the MA TA, a significant posttest difference was found for phase 1 (*t*_(18)_ = 2.660 and *p* = 0.012). For the LA FCR, phase 5 showed a significant posttest difference (*t*_(18)_ = 2.718 and *p* = 0.018), and, for the MA FCR, phase 1 showed a significant posttest difference (*t*_(18)_ = 2.660 and *p* = 0.012).

Investigating background EMG levels between tests allows for comparison of reflex amplitudes that cannot be explained by scaling with background EMG. Figure 8 shows bEMG (lines) during walking at all phases. For bEMG at specific phases of walking, there are significant differences between baseline and posttest values for the LA and MA FCR and LA PD muscles. For the MA FCR, significant differences were found for phases 2 (*t*_(18)_ = 2.227 and *p* = 0.036), 3 (*t*_(18)_ = 2.142 and *p* = 0.044), and 4 (*t*_(18)_ = 2.406 and *p* = 0.033). For the LA FCR, significant differences were found for phase 7 (*t*_(18)_ = 3.578 and *p* = 0.004). For the LA PD, significant differences were found for phases 1 and 2 (*t*_(18)_ = 2.407 and *p* = 0.033 and *t*_(18)_ = 2.754 and *p* = 0.017, resp.)

**Figure 9:**
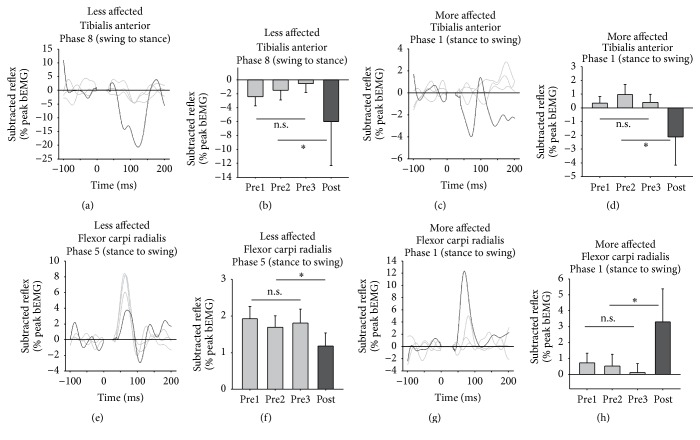
Normalized background EMG and reflex amplitudes at specific phase of interest during walking. Bar graphs are means (±standard error) for reflex amplitude averaged across all participants for baseline and posttest values. Line graphs are means (±standard error) for bEMG. ^*∗*^indicates significant differences between the pretest average and the posttest value. There were no significant differences for bEMG.

Figure 9 shows cutaneous reflexes during walking at specific phases of interest. Reflex modulation for the LA and MA TA and FCR is shown for specific phases of interest. At these phases, there are significant effects of training on posttest values and no significant differences in bEMG. Line graphs are of the subtracted reflex averaged across all participants for that phase.

## 4. Discussion

This project tested the efficacy of A&L cycling training for improving walking ability after stroke. Participants performed A&L cycling three times per week for five weeks for 30 minutes of exercise time each session. This aggregates 450 minutes of activity, performed at a moderate level, which improved walking after stroke. A&L cycling training improved clinical walking status, increased strength, range of motion, and temporal parameters of treadmill walking, and improved modulation of muscle activity and cutaneous reflexes. These results demonstrate that maximizing activity in inherent arm and leg connections spared after a stroke, with A&L cycling, could facilitate motor recovery. A&L cycling could be used as a novel rehabilitation modality to maximize functional motor recovery and improve walking ability after stroke.

A&L cycling training produced global changes in clinical status. There was a 4.19-second improvement in the Timed Up and Go test. This corresponds to a noticeable change in ability as the minimal detectable change for chronic stroke participants is 2.9 seconds [52]. Although there was an improvement in time taken for this test, values still fall below normative values for community-dwelling elderly people who finish the test in approximately 9–12 seconds [53]. For the 10 m walk test, speed increased by 0.06 m/s indicating a small but meaningful change [54]. Normative data for the stroke population (*n* = 48, age 68, with reduced muscle strength and walking capacity) is 0.84 ± 0.30 m/s. For the 6-minute walk test, participants improved by walking an additional 114.87 ft which is above a minimal detectable change of 112.76 ft for stroke participants [55]. Therefore, in summary, the walking tests showed minimal changes outside of error that reflect a true change between baseline tests and posttest values. For the balance test, scores on the Berg Balance Scale improved by 2.12 points which is just below the 2.5-point minimal detectable change criterion difference for a chronic stroke population [56]. We consider this change significant given that A&L cycling may not require the same trunk and pelvic control that walking does yet still improve balance after training.

Strength during isometric contractions increased for both the LA side and the MA side for plantarflexion and dorsiflexion following A&L cycling training. For the LA SOL and the MA TA, the increase in force was correlated with an increase in EMG. Handgrip strength also increased for both hands following training. A concomitant increase in EMG with force was not surprising as there is a linear correlation between the amplitude of EMG and the force produced during isometric contractions [57]. It is surprising however that no increase in EMG was recorded for the LA and MA FCR to match the increase in grip strength. Cocontraction of adjacent muscle groups, that were not being recorded, could account for this difference. Alternatively, perhaps no increase in EMG activity of these forearm muscles was observed because in some cases we used a hand brace to secure the weakened hand to the ergometer handle during training. Nevertheless, these results show an overall increase in strength resulting from A&L cycling training. A number of other studies also report improvements in strength following treadmill training interventions in those with spinal cord injury [4, 58–60]. Similar observations have been made in stroke and there is a positive correlation between strength gains and walking speed [61, 62]. Strength gains, an indirect result of A&L cycling training, likely contribute to the increase in walking ability seen here.

Changes in several variables measured during walking gauge training transfer effects following A&L cycling training. Variables include EMG modulation, kinematics, and stride parameters including duration, stance percentages, and frequency. For depth of modulation following A&L cycling training, the LA TA showed decreased modulation representing a smoothing out of dorsiflexor activity. Increased control to eliminate unwanted dorsiflexor activity is required to increase walking endurance in people with hemiplegia after stroke [63]. For the arms, in general, the arm muscles showed increased depth of modulation after training. An increased depth of modulation, indicating an increase in the amount of phasic activity, which more closely represents what is found in neurologically intact participants [38], could have been due to changes in weight support borne through the arms after training. The significant changes in modulation indices and the pattern of EMG activity for FCR and PD muscles appear different between the MA and LA sides. This may be important for the A&L-induced walking improvements observed here where increased modulation of these muscles may decrease exaggerated interlimb neural coupling producing a “flexor synergy” that has been previously reported after stroke [64].

Walking kinematics for all joints tested increased range of motion following training with an average increase of 22%. The transfer from A&L cycling training to improve walking kinematics is particularly interesting given kinematics are constrained on the A&L cycle ergometer [11]. Several variables related to walking parameters were also changed by A&L cycling training. Stride duration was decreased following training related to an increase in stride frequency. Within a stride for the LA side, it was found that stance duration decreased, while swing duration increased. These changes in swing and stance duration represent a more normal gait pattern [65]. Treadmill belt speed between pretests and the posttest was held constant for that participant and cannot be implicated as a source of the change in walking parameters seen here.

Changes in cutaneous reflex modulation were taken as a proxy of spinal plasticity arising from the A&L cycling training. Overall reflexes showed some improved modulation patterns following training. Cutaneous stimulation produced reflex effects in all muscles tested and is modulated during walking in a similar way to that found in neurologically intact participants [66]. By using an index of modulation, it is possible to see how the depth of reflex modulation changed with A&L cycling training. In the LA TA and LA PD, reflex modulation increased, representing an overall increase in the depth of modulation, perhaps due to increased access to these interlimb networks following training.

When examining the grand average reflex traces from cutaneous stimulation, activity in the LA TA is mainly suppressive, while the MA TA shows mainly facilitations (see Figure 7). This is in line with previous observations of cutaneous responses in the TA following stroke [69], where, on the MA side, the decreased influence of the corticospinal tract on reflex excitability, as a result of the stroke lesion, fails to produce the appropriate suppressions associated with normal reflex activity [67]. When examining specific functional phases for walking, adaptive plasticity was seen following training. Responses in the LA TA at phase 8, representing the swing to stance transition, showed increased inhibition. In the MA TA at phase 1, reflexes turned from facilitation to suppression following training. Normally at these phases, in neurologically intact participants, inhibitory responses are observed in the TA to aid with safe footfall allowing passive plantarflexion [48, 70–73] and the reemergence of end-swing suppressions following training reveals the normalization of reflexes as a result of A&L cycling training. In the arm muscles, stimulation following training produced decreased facilitation on the LA side and increased facilitation on the MA side, again representing a return to what one normally observes in modulation in these interlimb networks [38, 39].

Together these results demonstrate that adaptive plasticity in interlimb spinal networks is possible following rehabilitative training. It is unknown, however, how long these results persist and their functional implications. Further investigation of chronic plasticity in somatosensory pathways is warranted in order to fully understand motor adaptation to maximize functional recovery after neurological injury.

### 4.1. Task Transfer and Asymmetry of Changes between Sides

A bias between the observed training transfer effects between the LA and MA sides existed following A&L cycling training. A larger effect of strength gains following A&L cycling training was observed for the MA side for ankle dorsiflexion, plantarflexion, and handgrip. However, following training, an asymmetry was still observed between sides where MA posttest values were still below LA pretest values. An asymmetry in strength gains was also observed following treadmill aerobic exercise in patients with chronic hemiparesis following stroke where the greatest relative strength gains were seen in the MA limbs [74]. Asymmetry was also observed for EMG modulation following A&L cycling training where modulation was greater for the MA PD. The bias towards greater improvement on the MA side following A&L cycling training likely results from the increased potential for improvements on the MA side due to the higher degree of impairment [75]. Despite increased range of motion, alignment of walking kinematics between the LA and MA sides did not appear to improve after A&L cycling training. Therefore, although A&L cycling training does appear to result in a positive task transfer to improved walking, it does not produce a return to symmetry as kinematics on the MA side are still quite different from kinematics on the LA side.

A&L cycling training was an indirect training paradigm where walking was not the target of the training. The improvements in the trained task of A&L cycling transferred to improvements in the untrained task of walking. A few studies in stroke suggest partial transfer of a trained task on improving walking. For example, fitness training, high-intensity therapy, and repetitive task training all show beneficial improvements to walking after stroke [76–79].

The success of training transfer depended on how similar A&L cycling and walking are to each other and, indeed, all forms of rhythmic human movement share common neural elements [80]. The “common core” controlling cyclical limb movements is predicated upon multisegmental central pattern generating networks reinforced by somatosensory feedback regulated by supraspinal inputs [80–83]. Common neural elements are seen across different forms of walking (level, incline, and stair climbing; [46]), between different modes of rhythmic arm movement [9, 84], and between different modes of arm and leg coordination during recumbent stepping, cycling, and walking [85, 86]. The neuronal activity associated with generating rhythmic A&L cycling contains about 60% of what is found during treadmill walking [11] implying that rhythmic arm and leg movement performed in a task such as cycling could activate common locomotor networks.

Improvements in the temporal parameters of walking, kinematics, muscle modulation during walking, and clinical assessments of walking all demonstrate a positive transfer of A&L cycling training to enhanced walking function. The locus of task transfer is unknown but could originate from shared neural elements between the two tasks of A&L cycling and walking. More research on which physiological systems are affected by A&L cycling training is warranted.

### 4.2. Study Limitations

The observed improvement in walking could have been due to enhancements in cardiopulmonary fitness following A&L cycling training, a regular, prescribed fitness program. However, the level of training intensity for A&L cycling was quite low with little change in HR observed over a training session. The level of aerobic activity required to increase cardiopulmonary fitness in individuals with stroke is more intense than the level of exercise here [87, 88]. Future studies could, however, measure changes in cardiovascular function as a result of A&L cycling training. Another limitation of this study also has to do with the change in our intended sample. Although some participants did withdraw, significant effects were seen for many of the dependent variables indicating sufficient power. Additionally, intervention studies have often used reference untrained “control” groups to compare against the intervention or treatment groups. We have instead opted for the “multiple baseline” model where each participant serves as their own control and no committed volunteer participants are relegated to the role of an untrained control participant. In addition, in studying a patient population, there tends to be a large degree of between-subject variability as there is a wide range of abilities across participants. However, using a multiple baseline approach, we are able to mitigate this limitation as participants are instead compared against their own individual variabilities generated over multiple baseline sessions. We believe that multiple baseline measures should be considered a valid alternative or replacement to the concept of a control group.

### 4.3. Clinical Translation

Transfer of improvements following A&L cycling training to enhance walking could open the way to the development of a new approach for the rehabilitation of stroke patients. Current therapies for walking do not fully exploit the neuronal and mechanical linkages between the arms and legs that are inherent parts of human locomotion [6, 9–11]. We have shown here that A&L cycling improves walking ability after stroke and suggest that A&L cycling be used as an additional training modality for locomotor recovery. A&L cycling is a safe and low-stress activity and the linked cranks allow for physical assistance to the weakened limbs to encourage rhythmic coordination. In addition, A&L cycle ergometers are widely available in most gyms and recreation centers and are relatively cheap to access. This type of community-based exercise allows for equalization of opportunity for training with increased equipment access outside of major rehabilitation centers. Increasing the ease of training based upon a device that could be more readily used in therapy would directly impact the health and quality of life for those who have suffered a stroke. Given that other types of training, such as strength training or treadmill training, also improve walking, we do not suggest that A&L cycling training be used to replace these therapies. Instead, we suggest that this therapy be used as an adjunct modality to improve walking ability after stroke and may be particularly valuable as a bridging approach for those who initially lack strength and balance control for independent walking. To fully understand the relative benefits of A&L cycling training to other therapies, a randomized controlled trial should be conducted. In addition, given the link between the arms and the legs, examining the benefits of just arm cycling training on enhancing walking ability after stroke should also be conducted.

## 5. Conclusion

A&L cycling training improves walking ability after stroke. Results showed improved clinical walking status, increased strength, improved physical performance on the untrained task of walking, and improved reflex modulation especially in the leg muscles. These results suggest that A&L cycling training, an accessible and cost-effective training modality, could be used to improve walking ability after stroke.

## Figures and Tables

**Table 1:** Participant data and clinical assessment parameters.

*N*	Sex/age/MA	Modified Ashworth (ankle/knee)	FAC (/6)	Chedoke-McMaster (A/H/L/F)	Monofilament (hand/foot)	Reflexes (S1/L1)	Years since stroke
1	M/74/R	3/1+	4	2/2/3/2	J 4.31/J 4.31	3+/1+	5
2	F/70/R	0/0	5	7/5/7/7	J 4.31/J 4.31	2+/2+	2
3	F/45/R	1/0	5	5/5/6/5	F 3.61/J 4.31	0/0	7
4	M/59/R	2/0	5	2/2/4/2	T 6.65/J 4.31	3+/3+	3
5	M/82/R	0/1	3	4/6/6/5	UTF/UTF	3+/0	3
6	M/86/L	1+/0	5	7/7/6/5	J 4.31/T 6.65	0/0	4
7	F/80/R	0/0	5	3/5/5/5	J 4.31/J 4.31	0/0	6
8	M/59/R	2/1	5	5/5/5/4	T 6.65/T 6.65	3+/4+	11
9	M/74/R	1/1	5	6/5/6/5	J 4.31/F 3.61	3+/2+	6
10	M/47/L	4/2	4	2/1/2/2	T 6.65/T 6.65	4+/3+	6
11	M/69/L	2/3	4	2/2/3/2	T 6.65/T 6.65	3+/3+	5
12	F/72/R	2/2	3	2/3/2/3	UTF/J 4.31	1+/3+	6
13	M/59/L	1/1	6	6/6/6/4	J 4.31/J 4.31	3+/2+	5
14	M/56/L	1/1	5	1/1/4/2	T 6.65/T 6.65	3+/3+	8
15	M/77/L	2/2	3	4/5/5/3	UTF/T 6.65	3+/3+	8
16	F/63/L	1/2	5	2/2/3/4	T 6.65/K 4.56	3+/1+	13
17	M/71/R	1/2	4	3/2/4/4	F 3.61/J 4.31	2+/3+	6
18	M/62/R	1+/2	4	4/3/4/5	D 2.83/D 2.83	3+/3+	8
19	M/78/L	3/1+	4	3/3/4/4	T 6.65/T 6.65	0/0	29

MA, more affected; M, male; F, female; L, left; R, right; FAC, Functional Ambulation Category; A, arm; H, hand; L, leg; F, foot; UTF, unable to feel; S1, 1st sacral vertebrae; and L1, 1st lumbar vertebrae.

**Table 2:** Single-subject analysis.

Measure	Number of participants (out of 19) with significant changes after training
Strength	
LA plantarflexion	10
LA SOL	10
MA plantarflexion	10
MA SOL	8
LA dorsiflexion	11
LA TA	7
MA dorsiflexion	12
MA TA	11
LA grip	14
LA FCR	7
MA grip	17
MA FCR	8
Walking bEMG modulation index	
MA SOL	13
MA TA	9
LA SOL	10
LA TA	12
MA FCR	12
MA PD	11
LA FCR	13
LA PD	12
Walking kinematics	
LA ankle	9
LA knee	9
MA ankle	10
MA knee	11
Walking parameters	
LA stride duration	8
MA stride duration	9
LA stance duration	11
MA stance duration	10
LA swing duration	14
MA swing duration	10
LA stride frequency	8
MA stride frequency	8
Walking cutaneous reflex modulation index	
MA SOL	13
MA TA	10
LA SOL	13
LA TA	12
MA FCR	13
MA PD	12
LA FCR	9
LA PD	12

MA, more affected; LA, less affected; SOL, soleus; TA, tibialis anterior; FCR, flexor carpi radialis; PD, posterior deltoid; bEMG, background electromyography.
